# Immediate Effects of an Elastic Knee Sleeve on Frontal Plane Gait Biomechanics in Knee Osteoarthritis

**DOI:** 10.1371/journal.pone.0115782

**Published:** 2015-01-26

**Authors:** Raphael Schween, Dominic Gehring, Albert Gollhofer

**Affiliations:** Department of Sport Science, University of Freiburg, Schwarzwaldstrasse 175, D-79117 Freiburg, Germany; Duke University, Community Health and Family Medicine, UNITED STATES

## Abstract

**Introduction:**

Osteoarthritis of the knee affects millions of people. Elastic knee sleeves aim at relieving symptoms. While symptomatic improvements have been demonstrated as a consequence of elastic knee sleeves, evidence for biomechanical alterations only exists for the sagittal plane. We therefore asked what effect an elastic knee sleeve would have on frontal plane gait biomechanics.

**Methods:**

18 subjects (8 women, 10 men) with osteoarthritis of the medial tibiofemoral joint walked over ground with and without an elastic knee sleeve. Kinematics and forces were recorded and joint moments were calculated using an inverse dynamics approach. Conditions with sleeve and without sleeve were compared with paired t-Tests.

**Results:**

With the sleeve, knee adduction angle at ground contact was reduced by 1.9±2.1° (P = 0.006). Peak knee adduction was reduced by 1.5±1.6° (P = 0.004). The first peak knee adduction moment and positive knee adduction impulse were decreased by 10.1% (0.74±0.9 Nm•kg-1; P = 0.002) and 12.9% (0.28±0.3 Nm•s•kg-1; P < 0.004), respectively.

**Conclusion:**

Our study provides evidence that wearing an elastic knee sleeve during walking can reduce knee adduction angles, moments and impulse in subjects with knee osteoarthritis. As a higher knee adduction moment has previously been identified as a risk factor for disease progression in patients with medial knee osteoarthritis, we speculate that wearing a knee sleeve may be beneficial for this specific subgroup.

## Introduction

Knee Osteoarthritis (KOA) is a widespread degenerative joint disease whose prevalence increases with age [1,2] and which substantially impairs patients’ quality of life [3]. Therapeutic approaches for KOA include medication, exercise, and surgery. Additionally, knee braces, wedged insoles and elastic knee sleeves [4,5] are often applied in order to lessen symptoms and delay disease progression. Knee braces usually have a certain mechanical stiffness and are intended to selectively unload the affected compartment (in unicompartmental tibiofemoral KOA) by applying a valgus or varus moment to the knee, and to increase stability [5]. There are several studies showing biomechanical alterations with knee braces, especially reduced adduction moments with valgus bracing [6,7]. Knee sleeves are mostly elastic, and provide lower mechanical stability than braces. They have been shown to improve function [8–11] and quality of life [9], and to relieve pain [8] in patients with KOA. There are also reports of improved proprioception [12,13] and postural control [14] in KOA patients wearing knee sleeves.

However, evidence for the effects of knee sleeves on knee joint biomechanics is limited. To our awareness, there is currently only one study evaluating the effects of an elastic sleeve on gait biomechanics in KOA patients [15]. These authors investigated walking of patients with medial KOA at a self-selected “fast” pace in four different treatment conditions. They found a significant increase in knee flexion at ground contact and a significant decrease in initial loading rate and muscle co-contraction when a knee sleeve was worn [15]. It was concluded that decreases in sagittal loading rate could promote improvements in functionality and reduction of symptoms over time [15]. The study therefore demonstrates that an elastic knee sleeve can cause gait changes that may have positive effects for patients in the long term. However, its focus is limited to the sagittal plane, and we are not aware of any study that has investigated effects of an elastic knee sleeve on gait biomechanics in the frontal plane. This is surprising because previous research shows that knee adduction angles [16,17] and knee adduction moments [18] during gait are altered in KOA patients compared to uninjured subjects. Specifically, knee adduction angle and knee adduction moments were found to be increased in patients with medial KOA and decreased in lateral KOA [16–18]. Furthermore, these alterations appear to be highly relevant, as prospective studies have identified a higher adduction moment and adduction impulse as risk factors predicting disease progression in patients with medial compartment tibiofemoral KOA [19,20]. Taking these results into account, the potential effects of elastic knee sleeves on frontal plane gait biomechanics could be highly relevant for their therapeutic outcome. Therefore, an analysis of these effects seems warranted. For these reasons, our study was designed to investigate the immediate effects of an elastic knee sleeve on frontal plane gait kinematics and kinetics in subjects with medial KOA.

## Methods

Experiments were conducted in accordance with the Declaration of Helsinki (Seoul 2008) and were approved by the ethics committee of the University of Freiburg (107/12). All subjects gave written informed consent. All values are reported as mean±standard deviation if not indicated otherwise.

### Subjects

Eighty potential subjects were recruited through a report in a local newspaper and participated in an internet- and telephone-based pre-screening. Thereafter, 19 subjects were invited for and completed gait analysis at our laboratory. Inclusion criteria were KOA in one or in both legs with one leg being more pronounced (higher Kellgren-Lawrence grade; termed “affected” leg in the following) according to the most recent diagnosis by subjects’ respective physicians. In the affected leg, KOA had to be restricted to or most pronounced in the medial tibiofemoral compartment (medial KOA). Exclusion criteria were an age below 25 or above 65 years, neurological disorders, prosthetic implants in the hip, knee or ankle joint, and any condition contraindicating the physiological demands of the gait analysis. Age, weight, height, and Body Mass Index (BMI) were on average 50±9 years, 62±6 kg, 166±6 cm and BMI 23±2 for women, and 55±7 years, 87±16 kg, 181±8 cm and BMI 26±4 for men. One subject was diagnosed with an intermediate Kellgren-Lawrence grade of 1–2, the other subjects’ Kellgren-Lawrence grades ranged from 2 to 4 with a median of 3. All subjects completed the German version of the 12-item Oxford Knee Score [21] prior to testing. In 8 subjects the KOA was directly linked to a previous injury (secondary KOA). Four subjects had taken pain medication within 48 hours before testing. When asked about the quantity of their “sports activity” (note that subjects may have differed in their interpretation of this term), 14 subjects stated that it was 2 hours per week or more.

### Gait analysis

Subjects performed straight walks of about 10 meters length both with (sleeve condition; SL) and without (no-sleeve condition, NS) a commercially available elastic knee sleeve (GENUTRAIN 7, BAUERFEIND AG, Zeulenroda, Germany) worn at the affected leg. Order of conditions was randomized in order to prevent potential fatigue-related effects from biasing the analysis. The start point was adjusted so that subjects would hit a force plate (BP600900–2000, ADVANCED MECHANICAL TECHNOLOGY INC., Watertown, MA) with their affected leg. Subjects were unaware of the force plate and were told to look straight ahead in order to ensure a normal posture and gait pattern. A trial was considered valid if the force plate was hit fully and by one foot only. Additionally, walking speed was assessed with light barriers (TIMER S3, ALGE, Maienfeld, Switzerland) and had to be within a range of 0.06 m·s^-1^ in each direction around the subjects’ individual preferred walking speed. The latter was determined prior to testing by letting subjects perform several walks at a speed “at which you would go shopping” and was, on average, 1.4±0.14 m·s^-1^. In cases when subjects exceeded the speed range, they were told if they were too fast or too slow and the trial was dismissed. This procedure was repeated in each condition until 10 valid trials were captured. Subjects wore standardized shoes (SPEZIAL, ADIDAS, Herzogenaurach, Germany) with low cushioning and no custom insoles throughout the experiment to prevent potential footwear-related effects from biasing the analysis.

At the end of the session, subjects completed a final questionnaire, which included two visual analog scales (ranging from 0 to 10) asking if subjects felt any changes in pain or stability when wearing the sleeve (0 “pain increase” to 10 “pain reduction” and 0 “stability increase” to 10 “stability reduction”, 5 was “no change” in both cases).

### VICON

Kinematic analysis was performed at a sampling frequency of 200Hz using a 3D-motion analysis system (VICON V-MX, VICON MOTION SYSTEMS LTD., Oxford, UK). In order to assess joint kinematics, a cluster-based marker set was used, with retro-reflective marker clusters placed on pelvis, thigh, shank, and rear foot. Additional markers were attached to the lateral and medial epicondyle of the knee and to both malleoli in a static standing trial, which was used to calculate the segment lengths and joint centres, and to determine the segment coordinate systems in accordance with the standards of the International Society of Biomechanics [22,23]. Three-dimensional joint rotations were determined using Euler angles with a flexion/extension, adduction/abduction and internal/external rotation sequence. External joint moments were calculated in the distal segment coordinate system with a standard inverse dynamic approach integrating kinetic data from the force plate sampled at 1000Hz. Marker trajectories as well as ground reaction forces were low-pass filtered at 15Hz (Butterworth, 4^th^ order) prior to calculating joint angles and joint moments (relative to body weight). All calculations were performed in an integrated software package (BODYBUILDER 3.6, VICON MOTION SYSTEMS LTD., Oxford, UK). The joint angles and moments were calculated for each measured time frame, resulting in curves of joint angles or moments against time. Curve length was normalized to the duration of the support phase (= 100%) of the gait cycle as determined via the force plate to enable inter-individual comparisons. The curves were then averaged over the ten repetitions of each condition (SL / NS) for each subject, respectively.

### Data analysis

Based on the purpose of the study we selected the knee adduction angle at ground contact, the peak knee adduction angle and the first peak knee adduction moment, defined as the maximum single frame value of the first 50% of the gait cycle, as parameters for our study. It has recently been suggested that the analysis of knee adduction impulse in addition to peak knee adduction moment provides a more comprehensive understanding of medial knee joint loading than peak knee adduction moment alone [24]. Therefore we additionally calculated the positive knee adduction impulse, defined as the positive area under the knee adduction moment curve. While the data used for the analysis of joint angles and moments were normalized to support phase duration, the joint impulse is calculated based on the actual time of stance phase and is therefore reported as Nm·s·kg^-1^.

### Statistical analysis

Statistical analysis was performed with SPSS STATISTICS 21 (IBM, Armonk, NY, USA) and EXCEL 2010 (MICROSOFT, Redmond, WA, USA). The differences between any pair of NS and SL parameters did not differ significantly from normal distribution according to visual inspection and Shapiro-Wilk test. Pitman-Morgan test for homogeneity of variances in repeated measures [25] did not indicate any significant differences between the variances of any two corresponding NS and SL parameters.

Therefore, two-sided paired t-tests were used to test for significant differences between the NS and the SL condition of the respective parameters. *P*-values were Bonferroni-corrected to account for multiple testing. A calculation of effect size (Cohen’s d) was performed on these t-tests using G*POWER (VERSION 3.1.7) [26,27]. Because a change in positive knee adduction impulse could theoretically depend on both, reduced moments and decreased support phase duration, we performed an additional t-test (uncorrected) to test for significant differences in support phase durations between the NS and the SL conditions.

The results of the visual analog scales on pain and stability from the final questionnaire were not normally distributed. Therefore, Wilcoxon Signed Rank test was used to test for significant differences from the hypothetical median of 5 (i.e. “no change”).

We additionally tested for correlations between the subjective changes in pain and stability and the biomechanical alterations observed. For this purpose we calculated Pearson’s correlation coefficients and corresponding (uncorrected) *P*-values between the visual analog scores for pain and stability and the differences SL minus NS of the knee adduction angle at ground contact, the maximum knee adduction angle, the first peak knee adduction moment and the positive knee adduction impulse, respectively.

## Results

The dataset of one subject could not be analysed due to technical difficulties so that, eventually, the datasets of 18 subjects (8 women and 10 men) were analysed. Three additional subjects misinterpreted the visual analog scales and were excluded from the analysis of these.

**Figure 1:**
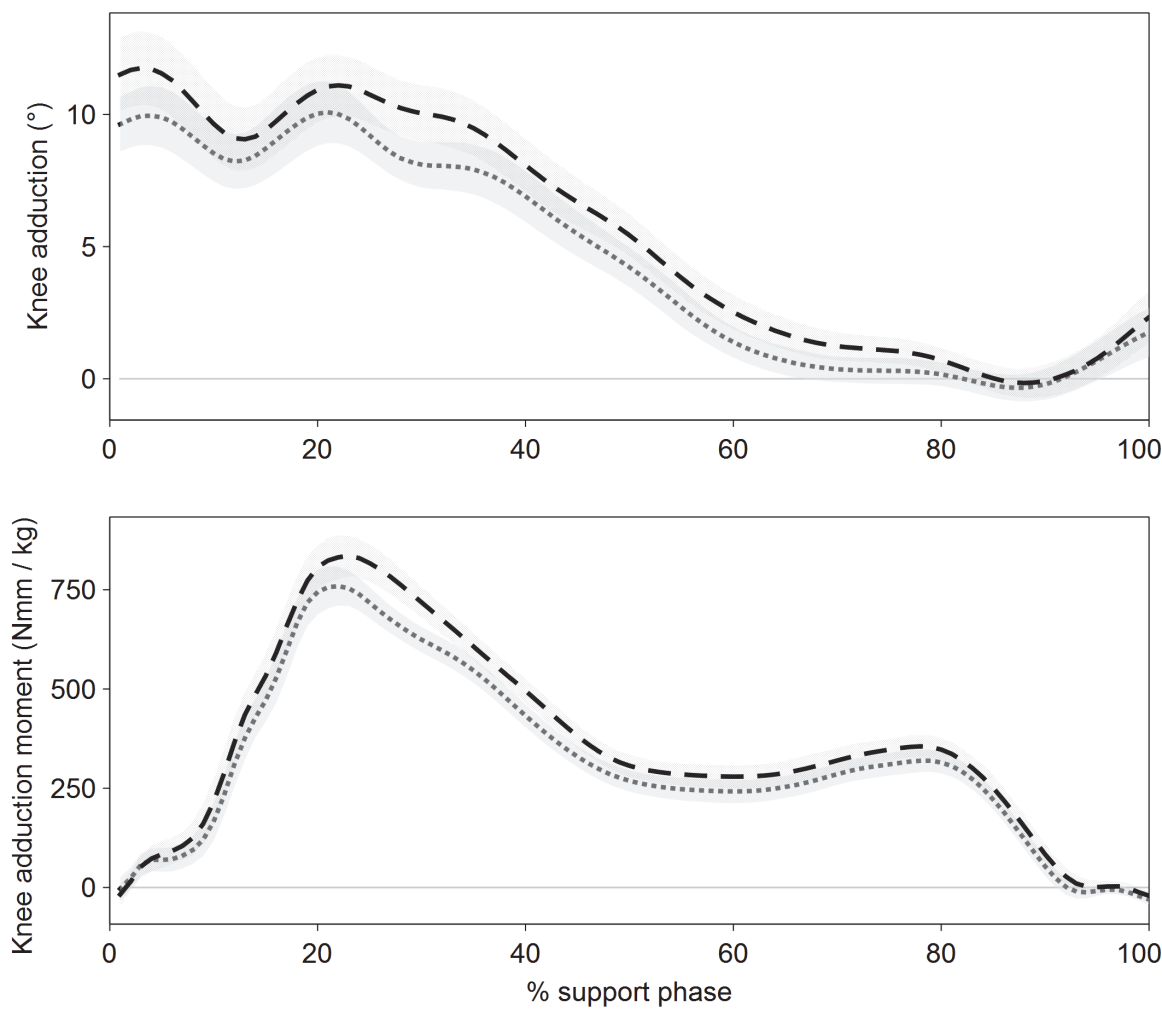
Knee adduction angle and external moment relative to the support phase of the gait cycle. Dashed lines and dotted areas represent mean and SD of all subjects (N = 18) for the condition without, dotted lines and grey areas for the condition with elastic knee sleeve.

Group means, standard deviations and effect magnitudes of kinematic and kinetic data are reported in Table 1. Knee adduction angles and moments were generally lower with the sleeve (Fig. 1).

**Table 1:** Results for kinematic and kinetic parameters.

	Without sleeve		With sleeve		Effect of sleeve	
	Mean	SD	Mean	SD	Mean	d
Knee adduction angle at ground contact (°)	11.5	7.8	9.6	8.3	- 1.9° *	0.79
Peak knee adduction angle (°)	14.1	6.4	12.6	6.7	- 1.5° *	0.57
First peak knee adduction moment (Nm·kg^-1^)	0.854	0.261	0.780	0.292	- 10.1% *	0.70
Positive knee adduction impulse (Nm·s·kg^-1^)	0.243	0.85	0.215	0.84	- 12.9% *	0.89

d is the effect size calculated with G*POWER (VERSION 3.1.7) [26] and corresponds to Cohen’s d [27].

* indicates that the difference between means is significant (*P* < 0.05).

Joint moments are external.

**Figure 2:**
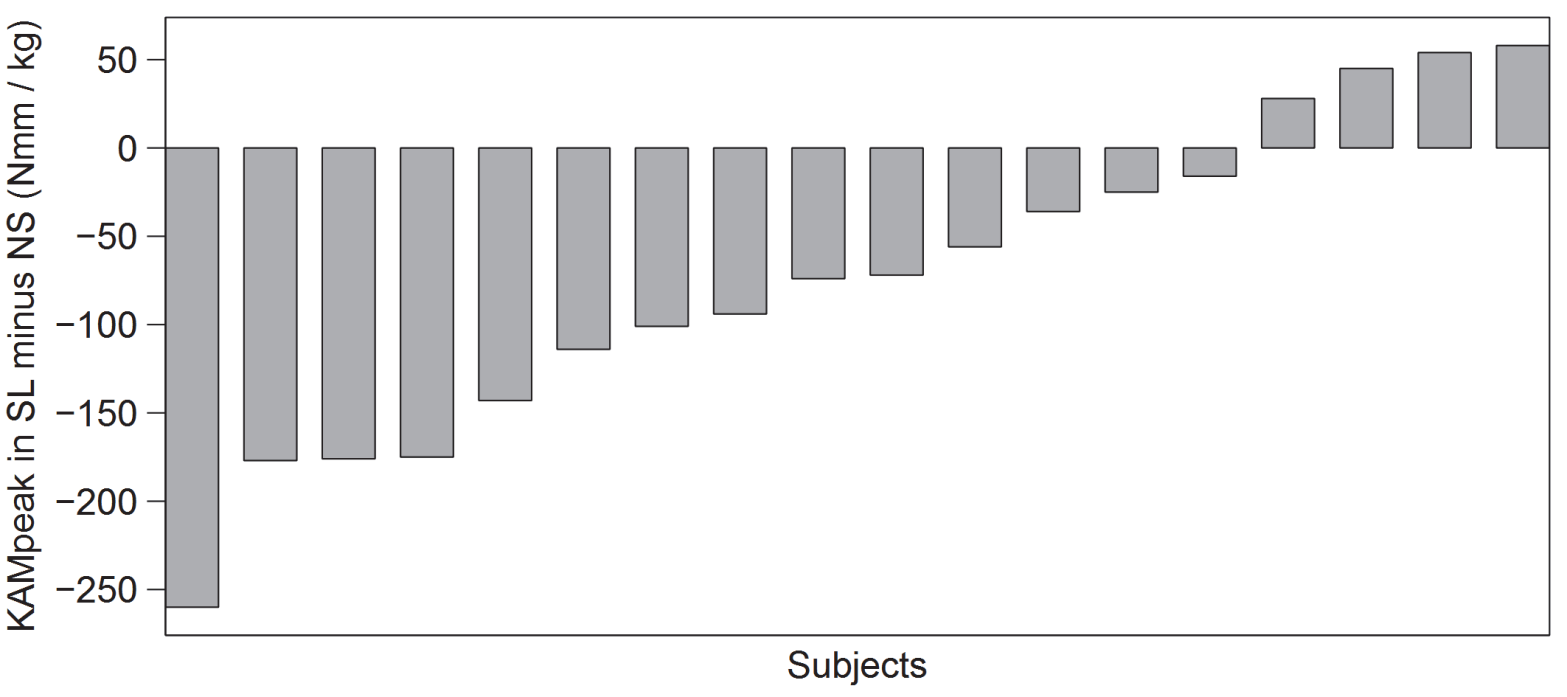
Differences between first peak external knee adduction moments relative to bodyweight (KAMpeak) in the condition without (NS) and with (SL) elastic knee sleeve for each subject (N = 18) (sorted from positive to negative for better overview).

On average, the knee adduction angle at ground contact was reduced by 1.9±2.1° (*P* = 0.006) while the peak knee adduction angle was reduced by 1.5±1.6° with the sleeve (*P* = 0.004). Fig. 2 provides a visual impression of the individual subjects’ development in peak knee adduction angles from the NS to the SL condition. Note that there were some subjects (4 out of 18) whose response to the sleeve was in a different direction than the group mean (Fig. 2). Kinetic analysis shows that the first peak knee adduction moment was on average reduced by 0.74±0.90 Nm·kg^-1^, which corresponds to 10.1% (*P* = 0.012) and the positive knee adduction impulse was reduced by 0.28±0.30 Nm·s·kg^-1^, which corresponds to 12.9% with the sleeve (*P* = 0.004). Support phase duration did not differ significantly between conditions (*P* = 0.64; mean duration: 0.67±0.06 s for each condition).

Subjects scored a median of 21 with a range from 14 to 40 out of 60 on the Oxford Knee Score. In the final questionnaire, the median value for the visual analog scales on the effects of the sleeve was 5.5 (range: 2.5 to 9.5) for pain, indicating a slight reduction of pain, and 2.5 (range 0 to 5) for stability, indicating an increase in stability. Both medians differed significantly (*P* = 0.034 and *P* < 0.001) from the hypothetical median of 5 according to Wilcoxon Signed Rank tests.

None of the correlations between subjective changes in pain and stability and biomechanical alterations approached significance. Detailed values are reported in Table 2.

**Table 2:** Results of the correlation analysis between subjects’ subjective evaluations of pain and stability change as measured by visual analog scales and the changes in the biomechanical parameters as derived by subtracting the value without sleeve from the value with sleeve.

	Perceived pain change		Perceived stability change	
	Pearson’s *r*	*P*	Pearson’s *r*	*P*
Change in knee adduction angle at ground contact	0.07	0.80	0.14	0.61
Change in peak knee adduction angle	0.01	0.97	- 0.03	0.92
Change in first peak knee adduction moment	- 0.21	0.45	0.36	0.19
Change in positive knee adduction impulse	- 0.18	0.52	0.20	0.48

Reported are Pearson’s correlation coefficients (r) and uncorrected P-values.

## Discussion

The present study shows that an elastic knee sleeve can influence frontal plane knee joint biomechanics in subjects with medial KOA. The sleeve in this study reduced knee adduction angles and moments through most parts of stance. Correspondingly, the positive knee adduction impulse was also reduced. As we found support phase durations to be very similar in the two conditions, we attribute this effect to the overall reduction in knee adduction moments rather than to a potential shortening of the support phase.

Knee adduction moments are thought to be positively correlated with medial contact forces in the knee [28] and longitudinal studies have identified a higher knee adduction moment [19] (but: [20]) and positive knee adduction impulse [20] as risk factors for disease progression in medial KOA. It can therefore be speculated that the decrease in knee adduction moments and impulse that we found with the sleeve in our study may lead to less disease progression over time. A possible decrease in medial contact forces could further offer one explanation for the positive effects of knee sleeves on pain and function that have been found previously.

With respect to the quantity of the effects, it is difficult to estimate what difference in knee adduction moments is needed for the change to be relevant for a therapeutic outcome. Miyazaki and colleagues [19] report a group mean of knee adduction moment for their subjects that is about 34% lower in the group without disease progression than in the group with disease progression. This interindividual difference is about 3 times as large as the −10.1% intraindividual difference in peak knee adduction moment observed in the SL compared to the NS condition in our study. However, these authors also report that only 4 out of 39 subjects with an adduction moment below 5% of bodyweight times size experienced disease progression, while 28 out of 35 subjects with a higher adduction moment did [19]. Further, the risk of disease progression was increased 6.46 times with a 1% increase in adduction moment [19]. Therefore small changes like the ones observed with the sleeve in our study may be relevant.

In this respect, it is also interesting to compare the effects of the sleeve in our study to the effects of braces and other treatments for KOA aimed at altering biomechanical properties. Draganich and colleagues [6] compared walking with an off-the-shelf and a custom made adjustable brace to unbraced walking in patients with medial KOA in a 4–5 weeks self-controlled intervention. They found a 4.3% reduction of knee adduction moment with the off-the-shelf brace and 14.4% for the custom brace (values calculated from reported data). Given the higher mechanical stability of braces compared to sleeves, it is surprising that the 10.1% reduction achieved with the sleeve in our study is higher than that of one of the two braces. Fantini Pagani and colleagues [7] compared walking of medial KOA patients without a brace to walking with a brace adjusted at 4° or 8° valgus and with laterally wedged insoles with an inclination of 4°. They found non-significant reductions of 2%, 7%, and 7% for the first peak external knee adduction moment with the 4° brace, 8° brace and the wedged insoles, respectively. These authors also compared the knee adduction angular impulse but, in contrast to our study, the normalization to support phase duration was not removed for its calculation [7]. For this parameter, they found a 14%, 18%, and 7% reduction with the 4° brace, 8° brace and wedged insoles, respectively. The 12.9% decrease observed for the sleeve in our study is thus slightly smaller than that of the two braces, but greater than that of the laterally wedged insoles. Further, the 10.1% decrease in first peak knee adduction moment is somewhat larger than that of the 8° brace and the wedged insoles and several times larger than the effect of the 4° brace on this parameter in the study of Fantini Pagani and colleagues [7].

Regarding the mechanisms through which the sleeve causes the observed effects, our study was not specifically designed to address these, largely due to the little prior knowledge about the effects of knee sleeves on KOA gait biomechanics per se. Therefore, our considerations on these mechanisms can only be of speculative nature. Due to the low mechanical stiffness of the sleeve, we assume that mechanical factors do not play a major role in the changes observed. Pain reduction also seems unlikely as a mediating factor, since it was only small (0.5 points median reduction on the 10 point visual analog scale of the final questionnaire), and because pain reduction has been associated with increased rather than decreased knee adduction in subjects with KOA in previous studies [29]. Another possible explanation could lie in the increased confidence in the stability of the knee that our subjects reported as an effect of the sleeve (2.5 points median increase on visual analog scale in final questionnaire). Many patients with KOA perceive instability (possibly as a consequence of impaired proprioception) of the knee and this could be a cause for typical alterations in gait patterns and could thus be reversed by the increase in stability.

None of the changes in our biomechanical parameters were significantly correlated with subjective ratings of pain or stability changes (cf. Table 2), which does not support a significant functional association between these parameters. This result should be interpreted cautiously as our sample size was rather small for inferential statistics on correlation coefficients (cf [30], p.116) and because there has been doubt with respect to retrospective evaluation of change as used for pain and stability assessment in this study [31]. Future studies may be well-advised to use pre-post measurement of subjective ratings of pain and stability. Nevertheless, as our current results do not support a significant role of perceived stability or pain, we speculate that the observed effects can be attributed to improved proprioception, which has been associated with knee sleeves before [12,13]. KOA has been associated with decreases in proprioception [13,32], and this lack of sensory information could be one of the causes for typical alterations in gait patterns, like increased adduction moments. Therefore, improved proprioception due to the sleeve may cause a revert to a more normal gait pattern. Further research is required to elaborate on these points. Specifically, future studies could address a possible correlation between proprioceptive improvements and biomechanical changes associated with an elastic sleeve.

The findings of this study are subject to some limitations. Subject selection was not designed to produce a representative sample. Therefore, our subject group may not be representative of the average KOA patient, as instantiated by the relatively low mean BMI of the female subjects. Further, in accordance with our inclusion criteria, all subjects analyzed suffered from osteoarthritis that was most pronounced in the medial compartment of one knee. It is thus important to note that patients with KOA different from this “medial” KOA in our study are likely to differ in their gait patterns [16,17] and therefore, their response to the sleeve requires further investigation. Further, recent findings indicate that knee alignment influences compensatory gait patterns in subjects with KOA [33]. Therefore, effects of sleeves may also depend on knee alignment. It seems advisable to assess this parameter in future studies. Further, our study only addresses short-term effects of the sleeve. Longitudinal studies would be useful to evaluate the long-term effects of knee sleeves on gait biomechanics. Our results may serve as a point of reference for the design of such studies. Finally, electromyographic measurement of muscle activity could further illuminate the mechanisms underlying biomechanical alterations, like they have for the sagittal plane [15,34]. However, because the skin areas covered by the sleeve conflict with proper electrode placement (SENIAM recommendations) [35], useful EMG measurements of the relevant muscles are difficult to obtain.

## Conclusion

To our awareness, this is the first study showing effects of an elastic knee sleeve on frontal plane gait biomechanics in (medial) knee osteoarthritis. Knee adduction angle and knee adduction moments were reduced with the sleeve. This could imply a positive effect of the sleeve for individuals with medial compartment tibiofemoral osteoarthritis. The persistence of this effect and its potential relation to clinical outcome should be further explored in longitudinal studies.

## Supporting Information

S1 Data csv-file containing the data underlying the analysis. (TXT)
